# Pseudomonas syringae pv. *phaseolicola* Uses Distinct Modes of Stationary-Phase Persistence To Survive Bacteriocin and Streptomycin Treatments

**DOI:** 10.1128/mBio.00161-21

**Published:** 2021-04-13

**Authors:** Ravikumar R. Patel, Prem P. Kandel, Eboni Traverso, Kevin L. Hockett, Lindsay R. Triplett

**Affiliations:** a Department of Plant Pathology and Ecology, The Connecticut Agricultural Experiment Station, New Haven, Connecticut, USA; b Department of Plant Pathology and Environmental Microbiology, The Pennsylvania State University, University Park, Pennsylvania, USA; c Center for Infectious Disease Dynamics, Pennsylvania State University, University Park, Pennsylvania, USA; d Huck Institutes for the Life Sciences, Pennsylvania State University, University Park, Pennsylvania, USA; University of Nebraska-Lincoln

**Keywords:** *Pseudomonas syringae*, persistence, persister, VBNC, streptomycin, tailocin, bacteriocin, viable but nonculturable

## Abstract

Antimicrobial treatment of bacteria often results in a small population of surviving tolerant cells, or persisters, that may contribute to recurrent infection. Antibiotic persisters are metabolically dormant, but the basis of their persistence in the presence of membrane-disrupting biological compounds is less well understood. We previously found that the model plant pathogen Pseudomonas syringae pv. *phaseolicola* 1448A (*Pph*) exhibits persistence to tailocin, a membrane-disrupting biocontrol compound with potential for sustainable disease control. Here, we compared physiological traits associated with persistence to tailocin and to the antibiotic streptomycin and established that both treatments leave similar frequencies of persisters. Microscopic profiling of treated populations revealed that while tailocin rapidly permeabilizes most cells, streptomycin treatment results in a heterogeneous population in the redox and membrane permeability state. Intact cells were sorted into three fractions according to metabolic activity, as indicated by a redox-sensing reporter dye. Streptomycin persisters were cultured from the fraction associated with the lowest metabolic activity, but tailocin persisters were cultured from a fraction associated with an active metabolic signal. Cells from culturable fractions were able to infect host plants, while the nonculturable fractions were not. Tailocin and streptomycin were effective in eliminating all persisters when applied sequentially, in addition to eliminating cells in other viable states. This study identifies distinct metabolic states associated with antibiotic persistence, tailocin persistence, and loss of virulence and demonstrates that tailocin is highly effective in eliminating dormant cells.

## Abstract (precis)

Populations of genetically identical bacteria encompass heterogeneous physiological states. The small fraction of bacteria that are dormant can help the population survive exposure to antibiotics and other stresses, potentially contributing to recurring infection cycles in animal or plant hosts.

## INTRODUCTION

The phenomenon of bacterial physiological tolerance to antibiotics is a long-standing problem in treating infection. The small fraction of cells in a bacterial population that survive after sustained lethal doses of antibiotics, termed persister cells, are a potential source of recurrent infections (1) or new resistance mutations (2, 3). Unlike genetically resistant cells, persisters occupy a low-metabolism state that is both nonheritable and reversible, where both growth and susceptibility are regained following antibiotic removal. The persister state may be induced in response to stress or stochastic variation in gene expression and occurs at increased frequency in stationary-phase populations due to nutrient starvation (4). Persistence is just one of several states of bacterial dormancy proposed in the literature (5), and researchers have observed phenotypic overlaps between persisters and viable-but-nonculturable (VBNC) cells, defined as living cells which are not revivable in standard media (6, 7). Single-cell observation after live/dead vitality staining has proven a useful strategy to phenotype heterogeneous populations. The redox indicator RedoxSensor green (RSG), a dye which fluoresces green when modified by bacterial reductases, is used to indicate the metabolic activity of live cells at the single-cell level; the fluorescence level is generally correlated with the activity of the electron transport chain and thus reflects respiration rate (8). Combining RSG with membrane permeability indicators, such as propidium iodide (PI), distinguishes dead cells from “low-redox” cells, i.e., dormant cells producing little-to-no metabolic reporter signal. Single-cell observation after RSG and PI staining was previously used to determine that both antibiotic persisters and starvation-induced VBNC cells occupy a state of low metabolic activity in Escherichia coli (9), and another live/dead cell-tracking approach determined that that VBNC cells outnumber persister cells after antibiotic treatment (10). The low metabolic rate of persisters and VBNC cells is thought to confer protection from antibiotics that target active processes (11).

Physiological tolerance may be an important issue in controlling bacterial plant diseases, which cause significant economic losses (12). Plant-pathogenic bacteria face a wide variety of stresses, including antimicrobial treatments, extremes in temperature, desiccation, nutrient starvation, and host redox defenses. VBNC cells are well documented in plant pathogens; nonculturable populations arise in response to plant or environmental conditions and can revive to initiate novel infections (13–17). The role of culturable persisters in plant disease is not well understood, but at least two phytopathogen species have been observed to form persisters to the aminoglycoside antibiotic streptomycin and to tetracycline (18, 19). Streptomycin and other antibiotics have been used for plant disease prevention since the 1950s and are still in regular use for a variety of crops in some parts of the world (20, 21). While relatively few countries monitor antibiotic use in plant production (22), global crop advisor records indicate that aminoglycosides are the most frequently applied antibiotic class (21). Streptomycin use in the United States has been largely restricted to managing pome fruit disease, but its use recently expanded after its authorization for use on citrus (23). The prevalence of antibiotic persistence and its potential impact on disease recurrence and management are important considerations in phytopathology.

A promising strategy for eradicating antibiotic persisters is combination treatment with compounds that attack static structures rather than growth processes, including membrane-disrupting polymyxin antibiotics, such as colistin (11). Candidate biological control treatments, including phage hydrolases, bacteriocins, and antimicrobial peptides, also disrupt bacterial membranes and are effective at killing antibiotic persisters (24–27). Bacterial subpopulations can also survive membrane-disrupting treatments in a conditional or nonheritable fashion (27–30). Investigating the frequency and biological basis of phenotypic tolerance to membrane disruptors will be important to understand the limits and extend the durability of these biocontrol strategies. Tailocins, or phage tail-like particles that disrupt the membranes of the target cell, are a class of bacteriocins with promise for highly specific control of plant disease (31). A tailocin purified from Pseudomonas syringae pv. *syringae* strain B728A efficiently kills the model bean pathogen P. syringae pv. *phaseolicola* strain 1448A (herein *Pph*; also referred to as P. savastanoi pv. *phaseolicola* in the literature). This and other tailocins prevent disease when applied prophylactically to plants (32–34). We recently found that a small fraction of the *Pph* population escapes lethal doses of tailocin via a nongenetic persister-like mechanism, and heritable tailocin resistance repeatedly arises in culture from the surviving population (32). We termed this “tailocin persistence” in light of its biphasic killing pattern.

In this study, we asked whether tailocin persistence in *Pph* is distinct from persistence to antibiotics, and hypothesized that tailocin persisters may be eliminated through combination therapy with antibiotics. We first established that *Pph* exhibits persistence to streptomycin and then used redox and membrane integrity reporter dyes to determine that tailocin treatment is much more efficient than streptomycin in rapidly eliminating viable cells from the unculturable population. Cell-sorting analysis determined that culturable tailocin persisters have a higher level of redox activity than those surviving streptomycin, and combination treatment with streptomycin and tailocin eliminated both culturable and nonculturable viable cells. Moreover, we found that culturable persisters to each treatment were able to infect host plants, while nonculturable redox-active cells were not. This study demonstrates a distinct physiological state of persistence in the presence of an effective membrane-disrupting biocontrol agent and establishes a foundation for future studies toward identifying persister eradication mechanisms.

## RESULTS

### Streptomycin and tailocin treatments yield similar frequencies of *Pph* persisters.

**FIG 1:**
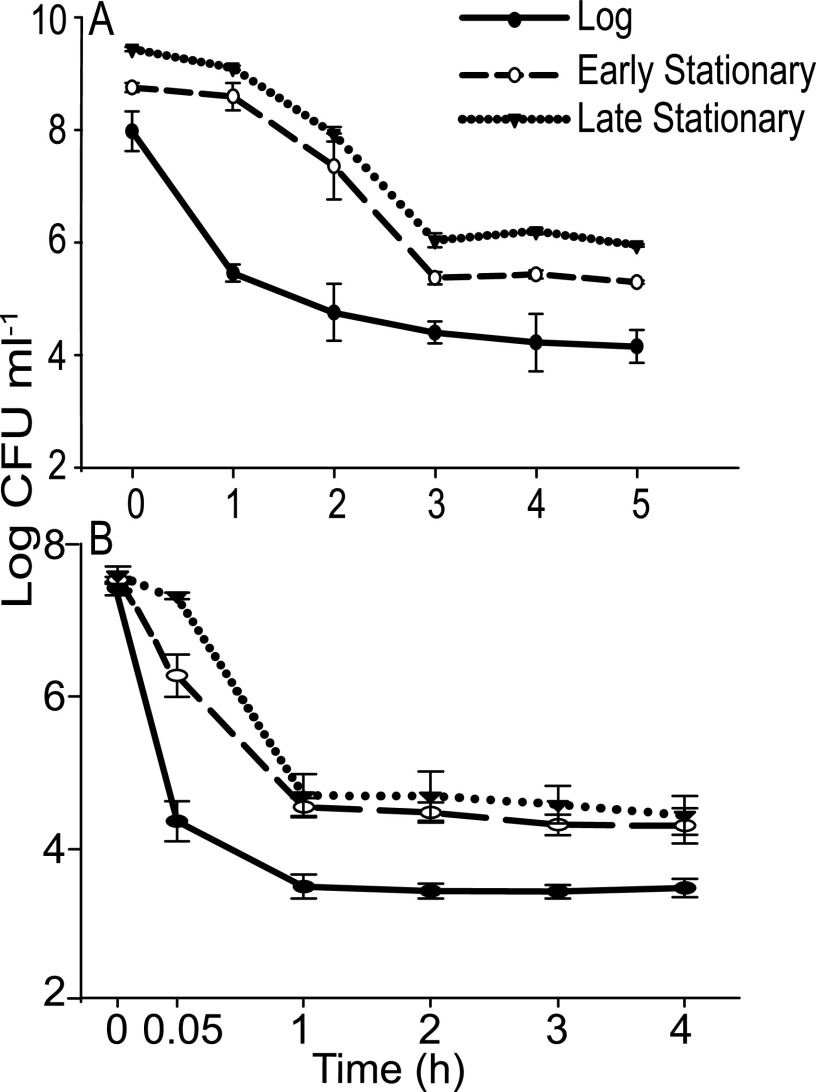
Comparison of persister frequencies after treatment of *Pph* with streptomycin (A) or tailocin (B). *Pph* cultures were treated in log phase, early stationary phase (20 h), or late stationary phase (96 h). (A) *Pph* cultures were treated with 5× MIC of streptomycin (16 μg ml^−1^), and culturable populations were enumerated hourly for 5 h. (B) Cultures were treated with 5× MIC of tailocin (250 AU) after resuspension in saline to ensure a standardized tailocin ratio per target cell, and culturable populations were enumerated immediately after tailocin addition (0.05 h or 3 min) and hourly. Error bars represent the standard deviations of the means from three replicate cultures. The experiment was performed three independent times.

We previously found that tailocin exposure results in a stable population of genetically susceptible *Pph* survivors, which we defined as tailocin persisters (32). In this study, we asked whether *Pph* exhibits persistence in the presence of the antibiotic streptomycin and whether streptomycin persistence is distinct from tailocin persistence. *Pph* growth curves were determined to establish the timing of the early and late stationary phases (see Fig. S1 in the supplemental material). Survival in the presence of 5× MICs of streptomycin and tailocin was characterized through kinetic killing curve assays at log phase, early stationary phase (20 h), and late stationary phase (96 h) (Fig. 1). In both early- and late-stationary-phase cultures, CFU counts declined to 0.04% of initial values within 3 h of streptomycin treatment, remaining stable at subsequent time points (Fig. 1A). Similarly, 0.06% of early- or late-stationary-phase *Pph* cells remained culturable after tailocin exposure, consistent with our previous observations, with the majority of killing occurring within a few minutes (Fig. 1B). Adding either treatment in log phase resulted in a lower proportion of survivors. For each assay, three colonies from the surviving stationary-phase populations were grown and retested to confirm that persistence rates at the final time point were not significantly different from that of the original culture (averaging 0.04% ± 0.00% and 0.06% ± 0.01% survival for streptomycin and tailocin, respectively). These results demonstrate that stationary-phase *Pph* populations form similar proportions of culturable persisters with both streptomycin and tailocin, although tailocin killing is far more rapid. Subsequent experiments in this study were performed on early-stationary-phase *Pph* unless otherwise noted, using the same doses and treatment durations as used for the experiments in Fig. 1.

10.1128/mBio.00161-21.1 FIG S1 Long-term growth curve of *Pph* 1448A. Overnight *Pph* cultures were inoculated 1/100 in fresh King’s B medium. CFUs were enumerated by plating cells on King’s B medium at the indicated time points up to 120 h. Download FIG S1, EPS file, 1.3 MB. Copyright © 2021 Patel et al. 2021 Patel et al. https://creativecommons.org/licenses/by/4.0/ This content is distributed under the terms of the Creative Commons Attribution 4.0 International license.

### Streptomycin and tailocin treatments have distinct effects on population physiology.

**FIG 2:**
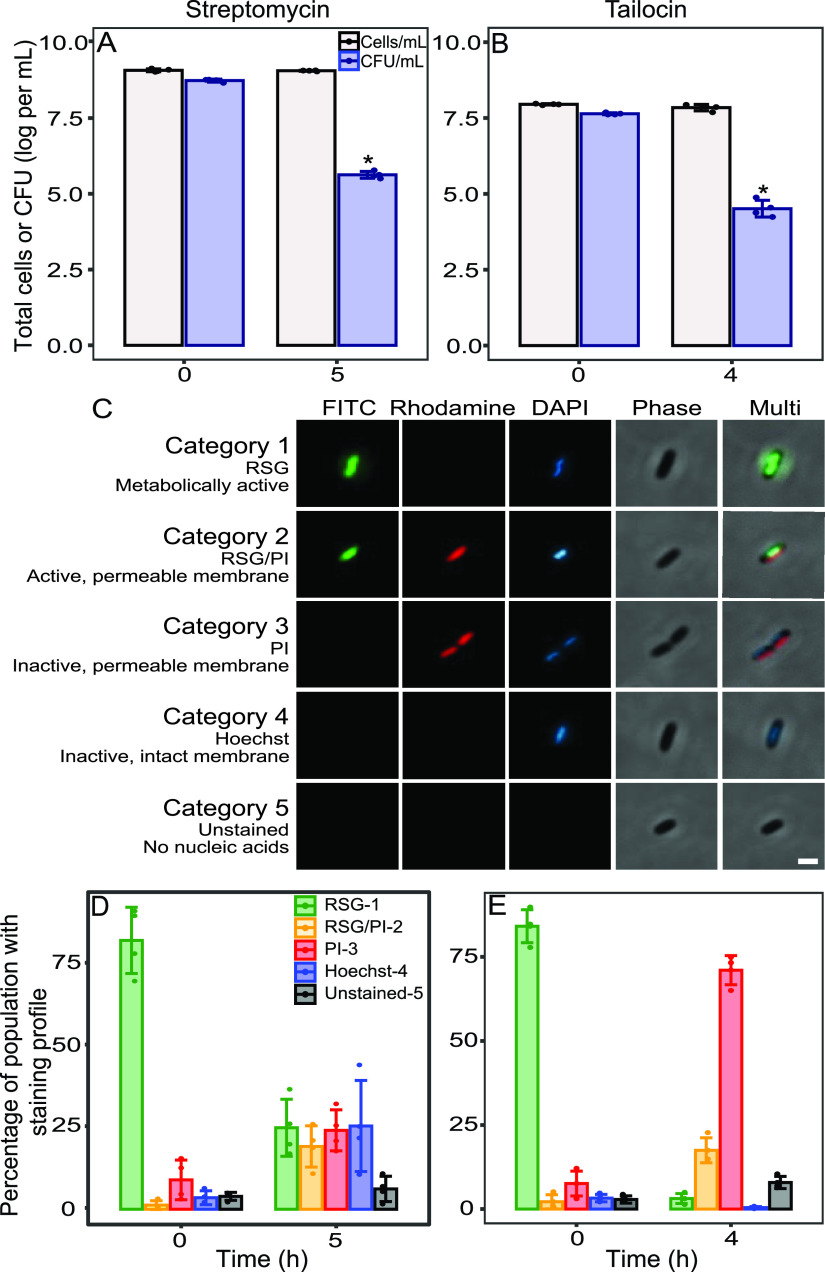
Distinct physiological states in stationary-phase *Pph* cultures after treatment with streptomycin or tailocin. Cultures were treated with streptomycin (A) or tailocin (B) and enumerated by hemocytometry (gray bars) or by dilution plating (blue bars). Asterisks represent a significant difference from the value at T0 (*P < *10^−6^). Tailocin-treated cultures averaged 20% fewer cells at 4 h (T4) than at T0, but the difference was not statistically significant (*P = *0.092). (C) Representative cells depicting *Pph* staining categories used in physiological profiling. Cultures were stained for 10 min with RSG, PI, and Hoechst 33342 and imaged on an agarose pad (1.5%). Rows depict single *Pph* cells imaged through three fluorescent filters, in phase contrast, and combined (40× objective). Images in the same row are taken from the same multichannel image of streptomycin-treated *Pph*. Scale bar = 2 μm. (D, E) Proportional compositions of *Pph* individuals in five physiological categories before and after treatment with streptomycin (D) or tailocin (E). Each data point represents the total proportion of all cells in a category at each culture and time point, counted across 10 images collected from three slides. A minimum of 650 cells were imaged per culture for tailocin at T4, and 1,000 cells were imaged for all other cultures and time points. Categories were assigned after visual inspection of each cell under three filters and in phase contrast. The four data points within a bar represent values from four independent experiments performed on different days. Bars represent the means and standard deviations across experiments.

Having established that both tailocin and streptomycin eliminate most culturable cells, we next sought to compare their efficiencies in eliminating the viable population, inclusive of nonculturable cells. We first measured treatment-induced changes in total cell concentration and culturable frequency. Hemocytometer readings revealed that streptomycin did not cause a reduction in the total concentration of cells in stationary phase or log phase (Fig. 2A; Fig. S2A). Tailocin caused a slight reduction in the number of cells, although this was statistically significant only in log phase (Fig. 2A; Fig. S2B). Dilution plating confirmed that treatments reduced the proportion of total stationary-phase cells (measured with a hemocytometer) that were culturable from 46% to 0.04% for streptomycin (Fig. 2A) and to 0.05% for tailocin (Fig. 2B), consistent with our earlier measures of survival over that of the time zero (T0) population (Fig. 1). To compare the physiological states of streptomycin- and tailocin-exposed *Pph* populations, we imaged cells on an agarose pad after staining them with a combination of three fluorescent dyes: the green vitality indicator RSG, the red membrane permeability indicator PI, and the blue membrane-permeant nucleic acid stain Hoechst 33342. This strategy allowed imaged cells to be classified into five categories (Fig. 2C): category 1, redox-active cells with intact membranes (green/blue) and with active reductase activity, reflecting active electron transport and respiration; category 2, redox-active cells with compromised membranes (green/red/blue); category 3, redox-inactive cells with compromised membranes (red/blue); category 4, redox-inactive cells with intact membranes and retention of nucleic acids (blue); and category 5, unstained “ghost” cells with no nucleic acid content, visible in phase-contrast only. Because PI stains only cells containing nucleic acids, the membrane status of category 5 cells is unknown. The method was first tested on log-phase and ethanol-killed cells to rule out signal interference or overlap between stains (Fig. S3), and we confirmed that the staining combination did not change the number of culturable cells in *Pph* samples (data not shown). In preliminary experiments, we noted that all *Pph* cells showed permeabilization and loss of redox activity starting after 2 h on the agarose pad; thus, all imaging was performed within 20 min after placement on the pad.

10.1128/mBio.00161-21.2 FIG S2 Proportional staining categories of log-phase *Pph* cultures after treatment with streptomycin or tailocin. (A, B) Concentration and culturability effects. Cultures were treated with streptomycin (A) or tailocin (B) and enumerated by hemocytometry (gray bars) or by dilution plating (blue bars). Asterisks represent a significant difference from the T0 value (*P < *0.005). CFU counts in both untreated cultures averaged 95% of hemacytometer-based cell counts, indicating the culturability rate in log phase. Tailocin-treated cultures averaged 54% fewer visible cells at T4 than at T0. (C, D) Proportional composition of *Pph* individuals in five physiological categories before and after treatment with streptomycin (C) or tailocin (D). Categories were assigned after visual inspection of each cell under three filters. Each data point represents 1,000 *Pph* cells counted across 10 images taken from at least three slides. The four data points for each treatment represent values from four independent experiments, and bars represent the means and standard deviations across experiments. Download FIG S2, EPS file, 1.8 MB. Copyright © 2021 Patel et al. 2021 Patel et al. https://creativecommons.org/licenses/by/4.0/ This content is distributed under the terms of the Creative Commons Attribution 4.0 International license.

10.1128/mBio.00161-21.3 FIG S3 Testing of a triple physiological staining method. Log-phase cultures of *Pph* were imaged with no treatment (top) and after ethanol treatment (bottom) after simultaneous staining with RSG, PI, and Hoechst 33342 of exponential-phase and ethanol-treated *Pph* cells. Images were collected through FITC, rhodamine, DAPI, and phase-contrast channels and combined into a multichannel image. Scale bar = 20 μm. Download FIG S3, TIF file, 3.0 MB. Copyright © 2021 Patel et al. 2021 Patel et al. https://creativecommons.org/licenses/by/4.0/ This content is distributed under the terms of the Creative Commons Attribution 4.0 International license.

In untreated stationary- and log-phase cultures, over 80% of the population was composed of redox-active, or category 1, cells (Fig. 2D and E; Fig. S2C and D; Table S1). After streptomycin treatment, log-phase *Pph* cultures were evenly distributed through categories 2 to 4, each comprising an average of 19 to 25% of the population (Fig. 2D). Log-phase cultures showed a similar diversification, although with a higher proportion of cells remaining in category 1 (Fig. S2C). Unlike streptomycin, tailocin treatment converted most of the stationary-phase population to category 3 (membrane compromised, metabolically inactive) within 3 min (Fig. S4). After 4 h, only 3% of the remaining cells were in category 1 (Fig. 2E; Table S1), and category 4 cells were extremely rare. Tailocin-induced changes in log-phase cultures were similar to those in stationary-phase culture, although there was a reduced proportion of redox-active category 2 cells after tailocin treatment compared to the log-phase results (Fig. S2D).

10.1128/mBio.00161-21.4 FIG S4 Stationary-phase *Pph* cultures stained with RSG, PI, and Hoechst 33342 before (A), 3 min after (B), and 4 h after (C) tailocin treatment. Scale bar = 20 μm. Download FIG S4, TIF file, 2.8 MB. Copyright © 2021 Patel et al. 2021 Patel et al. https://creativecommons.org/licenses/by/4.0/ This content is distributed under the terms of the Creative Commons Attribution 4.0 International license.

10.1128/mBio.00161-21.9 TABLE S1 Percentages of imaged log-phase and stationary-phase *Pph* cells in five fluorescent staining categories before and after antimicrobial treatment. Download Table S1, DOCX file, 0.01 MB. Copyright © 2021 Patel et al. 2021 Patel et al. https://creativecommons.org/licenses/by/4.0/ This content is distributed under the terms of the Creative Commons Attribution 4.0 International license.

Streptomycin-treated populations were nearly half comprised of redox-active cells with permeable membranes (category 2) or redox-inactive cells with intact membranes (category 4). These are two states not distinguished by common live/dead staining methods, so we investigated their properties further using image analysis. First, we asked whether activity was diminished in live membrane-compromised cells compared to that of cells with intact membranes and compared green signal intensities between the two phenotypes. Category 2 cells had a significantly reduced average RSG intensity compared with that of cells in category 1, indicating that membrane damage accompanies a loss of metabolic activity (Fig. S5A). Additionally, previous work found that starvation-induced persister and VBNC cells of E. coli occupy a state similar to category 4: they were metabolically inactive, were PI impermeable, and retained cell contents (7). Persister and VBNC cells were also characterized by an increased roundness that progressed to a spherical shape over time (7), and we next performed cell shape analysis to determine whether the roundness phenotype was also shared by category 4 cells of *Pph*. Category 4 cells had significantly increased average roundness compared with that of the four other categories of treated *Pph* cells (Fig. S5B), further demonstrating that the category 4 cells are phenotypically consistent with reported qualities of persistence. Together, the results indicate that streptomycin treatment shifts the majority of the *Pph* population into diverse physiological states, while tailocin treatment rapidly compromises redox activity and membrane integrity in the vast majority of the population. They also show that the proportions of redox-active and intact cells in either treated population (Fig. 1C and D; Table S1) far exceed the 0.04 to 0.05% rate of culturable persisters (Fig. 2A and B).

10.1128/mBio.00161-21.5 FIG S5 Intensity and roundness associated with physiological staining categories. (A) Average RSG signal intensities of *Pph* cells in the category 1 (redox-active) and category 2 (redox-active, membrane-permeable) staining categories. One hundred imaged cells were selected randomly from each of categories 1 and 2. Cells were selected from across four images representing four independent streptomycin-treated stationary-phase cultures. The intensity of the selected cells in the FITC channel was analyzed using the MicrobeJ plugin of Fiji. (B) Roundness of 100 randomly selected cells from each of the staining categories observed after streptomycin treatment, with the exception of category 5, for which only 70 cells could be observed. Roundness was analyzed in the phase-contrast images using the MicrobeJ plugin. Download FIG S5, EPS file, 2.5 MB. Copyright © 2021 Patel et al. 2021 Patel et al. https://creativecommons.org/licenses/by/4.0/ This content is distributed under the terms of the Creative Commons Attribution 4.0 International license.

### Streptomycin- and tailocin-culturable persisters occupy distinct physiological states.

While microscopic studies were useful for profiling redox and permeability changes following either treatment, this approach could not determine the culturability of each staining category. Therefore, we applied fluorescence-assisted cell sorting (FACS) to determine whether the culturable and infectious fractions of streptomycin- and tailocin-treated populations could be separated according to redox staining characteristics. Because propidium iodide had stained some redox-active *Pph* cells (i.e., category 2 cells), the propidium iodide appeared to be leaking into metabolically active cells. We sought an alternate permeability stain that could provide a distinct live/dead separation in two-color sorting studies. DRAQ7 is a far-red membrane-permeant dye that has been validated in eukaryotic cell culture studies (35) but is not widely used for determining viability in bacteria. In microscopic analysis on *Pph*, DRAQ7 stained redox-inactive cells, but unlike PI, was not observed to costain with RSG (Fig. S6). This indicated that DRAQ7 does not permeate cells with redox activity. A triple-staining experiment using DRAQ7 instead of PI was performed on treated and untreated *Pph* to confirm that the stain yielded estimates of redox-inactive membrane-compromised cells similar to those determined with PI staining (Table S2).

10.1128/mBio.00161-21.6 FIG S6 DRAQ7 staining is limited to redox-inactive cells. (A) Representative multichannel image of streptomycin-treated *Pph* cells after simultaneous staining with RSG, PI, and DRAQ7 for 10 min. Scale bar = 20 μm. (B) Three single *Pph* cells imaged in three fluorescence channels. Scale bar = 2 μm. Download FIG S6, TIF file, 2.9 MB. Copyright © 2021 Patel et al. 2021 Patel et al. https://creativecommons.org/licenses/by/4.0/ This content is distributed under the terms of the Creative Commons Attribution 4.0 International license.

10.1128/mBio.00161-21.10 TABLE S2 Percentages of imaged cells stained with RSG, DRAQ7, or Hoechst dye only in a microscopic analysis of stationary-phase *Pph* cells before or after antimicrobial treatment. Download Table S2, DOCX file, 0.01 MB. Copyright © 2021 Patel et al. 2021 Patel et al. https://creativecommons.org/licenses/by/4.0/ This content is distributed under the terms of the Creative Commons Attribution 4.0 International license.

**FIG 3:**
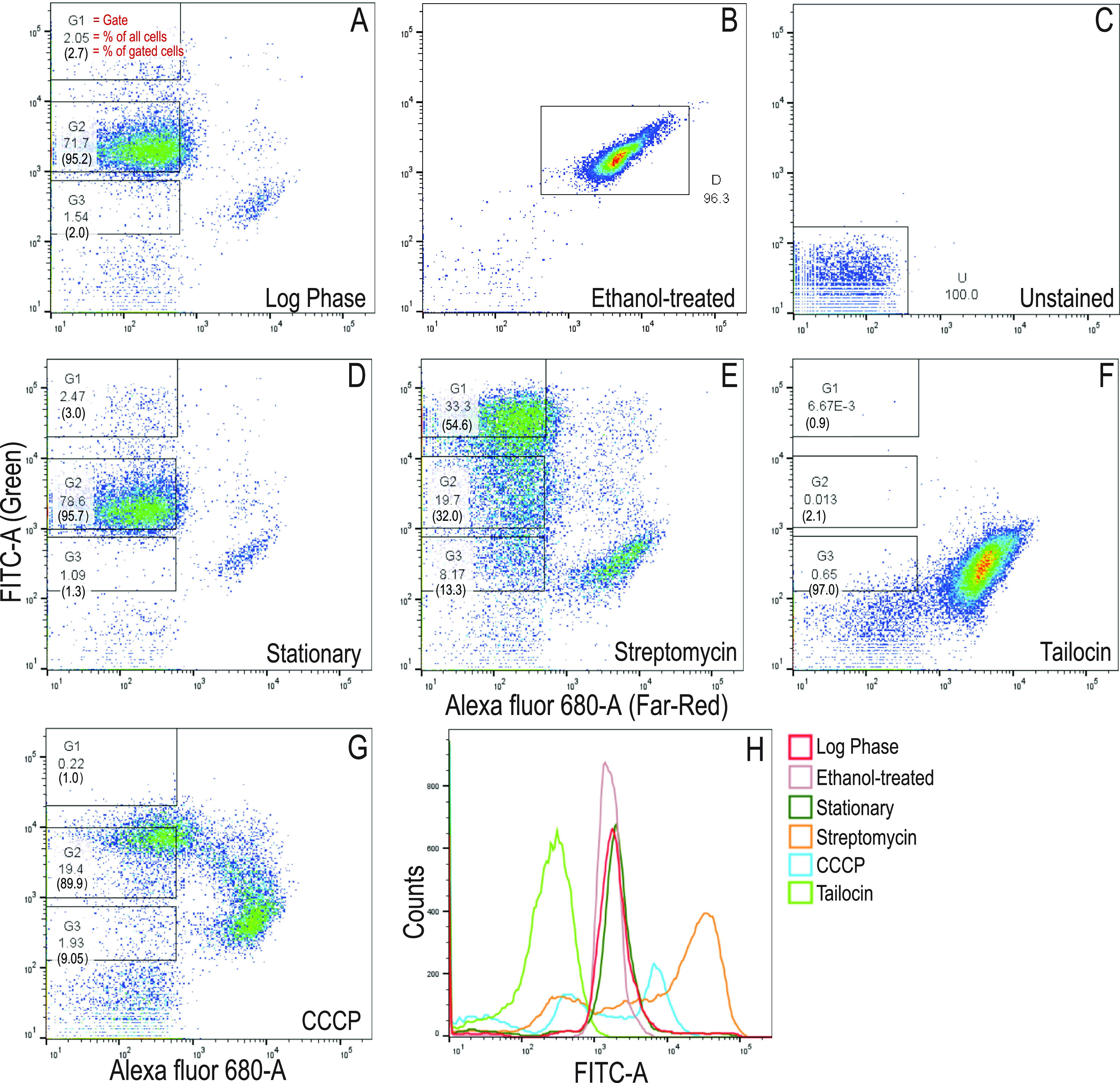
Flow cytometric analysis and sorting of *Pph* populations. (A to G) Density dot plots of RSG- and DRAQ7-stained *Pph* cultures in log phase (A), after treatment with ethanol (B), and without fluorescent stain (C) and in stationary-phase cultures with no treatment (D) or after treatment with streptomycin, tailocin, or CCCP (E to G). Each dot plot represents 50,000 sorted events. Within the DRAQ7-unstained fraction, three gates were assigned according to green fluorescence intensity above unstained-cell levels, and these were designated G1 to G3 (boxes in panels A and D to G). *x* and *y* axes are in relative fluorescence units. Numbers in gated boxes show the percentages of total cells found within the gate, followed in parentheses by the percentage of total gated cells found within that gate. (H) Histogram showing the distribution of green fluorescence intensity of populations plotted in panels A to G. The figure represents experiment 1 of the two independent experiments depicted in Fig. 4.

Flow cytometric analysis of untreated log- and stationary-phase, ethanol-killed, and unstained cells identified clear patterns associated with death and active growth (Fig. 3A to D). Consistently with microscopic observations, streptomycin treatment resulted in an apparent increase in both permeabilized cells and low-redox intact cells but also resulted in a large population of cells with an elevated redox signal (Fig. 3E). As expected, tailocin treatment permeabilized all but a small fraction of cells to DRAQ7 (Fig. 3F). To determine how the streptomycin-induced changes compared with the effects of a validated persister induction treatment, we also treated stationary cells with the protonophore carbonyl cyanide *m*-chlorophenylhydrazone (CCCP), a highly efficient inducer of multidrug-tolerant persisters in Pseudomonas aeruginosa (36). A killing curve assay demonstrated that 3 h of CCCP treatment (5× MIC, or 100 μg ml^−1^) resulted in a stable culturable population representing 13% of the initial count (Fig. S7). CCCP-treated cultures showed slightly increased RSG staining in intact cells, with many cells permeabilized to DRAQ7 (Fig. 3G). Histogram analysis supported the finding that streptomycin and CCCP treatments generated populations with increased redox signals, while tailocin treatment largely abolished redox activity (Fig. 3H). For unknown reasons, ethanol-killed cells had a higher level of green fluorescence in cell sorting than tailocin-permeabilized cells (Fig. 3H).

10.1128/mBio.00161-21.7 FIG S7 Persistence curve for CCCP. *Pph* cultures were treated with 5× MIC of CCCP (100 μg ml^−1^), and culturable populations were enumerated hourly for 5 h. Error bars represent the standard deviations of the means from three replicate cultures. The experiment was performed three independent times. Download FIG S7, EPS file, 1.3 MB. Copyright © 2021 Patel et al. 2021 Patel et al. https://creativecommons.org/licenses/by/4.0/ This content is distributed under the terms of the Creative Commons Attribution 4.0 International license.

**FIG 4:**
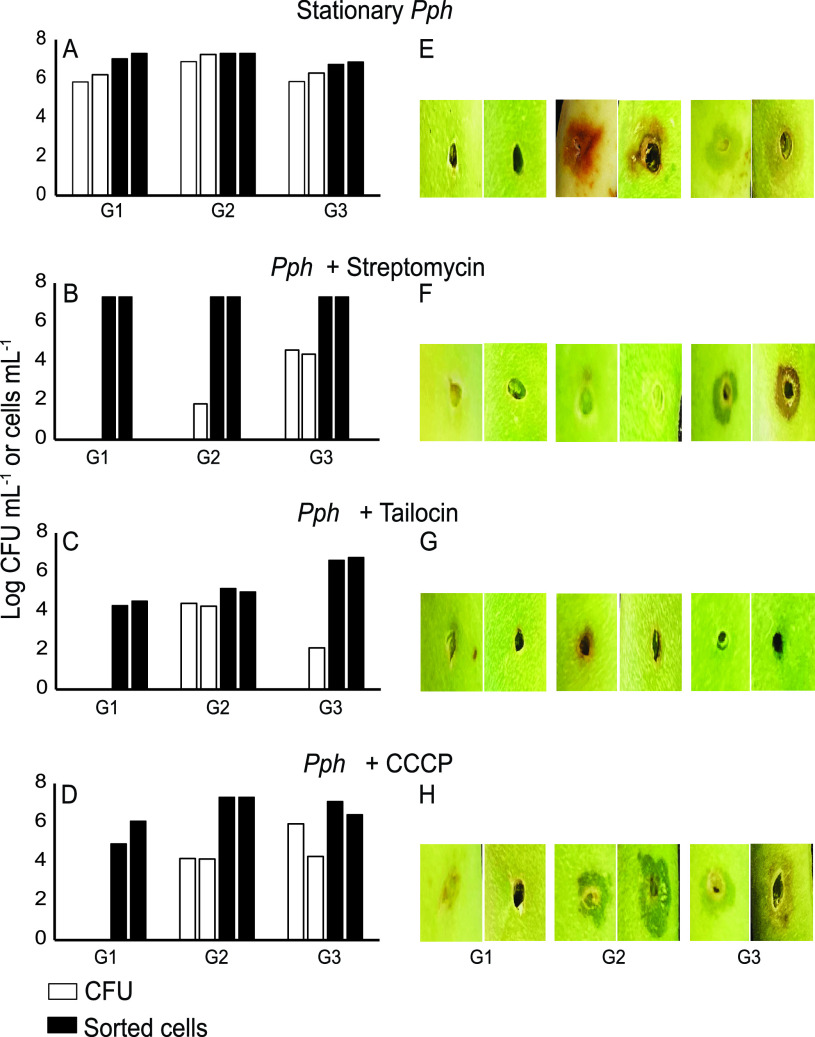
Culturable and infectious streptomycin- and tailocin-treated cells have distinct redox staining intensities. (A to D) Culturability of cells in RSG intensity gates G1 to G3. Black bars indicate total cells collected, and white bars represent numbers of CFU per milliliter, enumerated from the sorted fraction. The two bars in each sorted gate represent values from independent experiments 1 (left bar) and 2 (right bar). Samples were plated after sorting from stationary-phase *Pph* cultures without treatment (A) or after treatment with streptomycin (B), tailocin (C), or CCCP (D). (E to H) Symptom development on bean pods after wounding and inoculation with 30 μl of the sorted fraction after a 20× concentration was obtained. Images were taken 5 days after inoculation. Left and right images correspond to independent experiments 1 and 2, respectively.

Cells were separated according to physiological state to determine the culturability of each fraction. Optimization assays confirmed that no culturable cells could be recovered from DRAQ7-staining fractions, so we focused on the region of low DRAQ7 intensity. Cells were gated into fractions G1, G2, and G3, corresponding to the highest to lowest green fluorescence intensities (Fig. 3A and D to G). Cells intact after streptomycin, tailocin, and CCCP treatments fell primarily into the G1, G3, and G2 gates, respectively (Fig. 3E to G). Cells collected from each sorting gate were plated on culture media. In untreated cultures, colonies were recovered from all fractions (Fig. 4A). After streptomycin treatment, over 99.5% of colonies recovered came from the low-redox G3 fraction (Fig. 4B), even though this fraction represented only 13% of the intact cells that were gated (Fig. 3E). In contrast, in the tailocin-treated culture, over 99% of colonies were recovered from the G2 fraction (Fig. 4C), despite this fraction containing only 2% of the total gated, intact cells (Fig. 3F). Colonies were cultured from both the G2 and G3 fractions after CCCP treatment (Fig. 4D), but as with the other two treatments, nothing was cultured from the G1 fraction. These results demonstrate that the cells that are culturable after streptomycin treatment occupy a low-redox state consistent with dormancy, whereas those that persist after tailocin treatment are associated with a state of moderate redox activity. Additionally, diverse treatments resulted in a lack of culturability in cells with a high level of redox activity.

We next asked whether the culturability or redox activity of sorted fractions was associated with the infectious capacity of the pathogen. Due to the low volume of the sorted inoculum, the pathogenicity of the fractions was assessed in a qualitative bean pod inoculation assay, and symptoms of water soaking or necrosis were observed after 5 days (Fig. 4E to H). No symptoms developed after inoculation from the high-redox G1 fraction of any culture, even without antimicrobial treatment (Fig. 4E). For cultures treated with streptomycin, tailocin, or CCCP, symptoms were observed at sites inoculated with any fraction with a significant culturable population (∼10^4^ or greater CFU ml^−1^) (Fig. 4F to H). Symptoms were weakest in the tailocin-treated cultures, but this may be attributable to the low number of culturable cells obtained through sorting. Notably, for streptomycin- and tailocin-treated cultures, the fractions associated with the largest numbers of membrane-intact cells (G1 and G3, respectively) did not cause symptoms (Fig. 3E and F and Fig. 4F and G). These results demonstrate that culturability in media is associated with infection capacity in antimicrobial-stressed *Pph* and that the highest redox fractions of all cultures were noninfectious.

### Streptomycin- and tailocin-treated cells colonize the host at the same rates as untreated cells.

**FIG 5:**
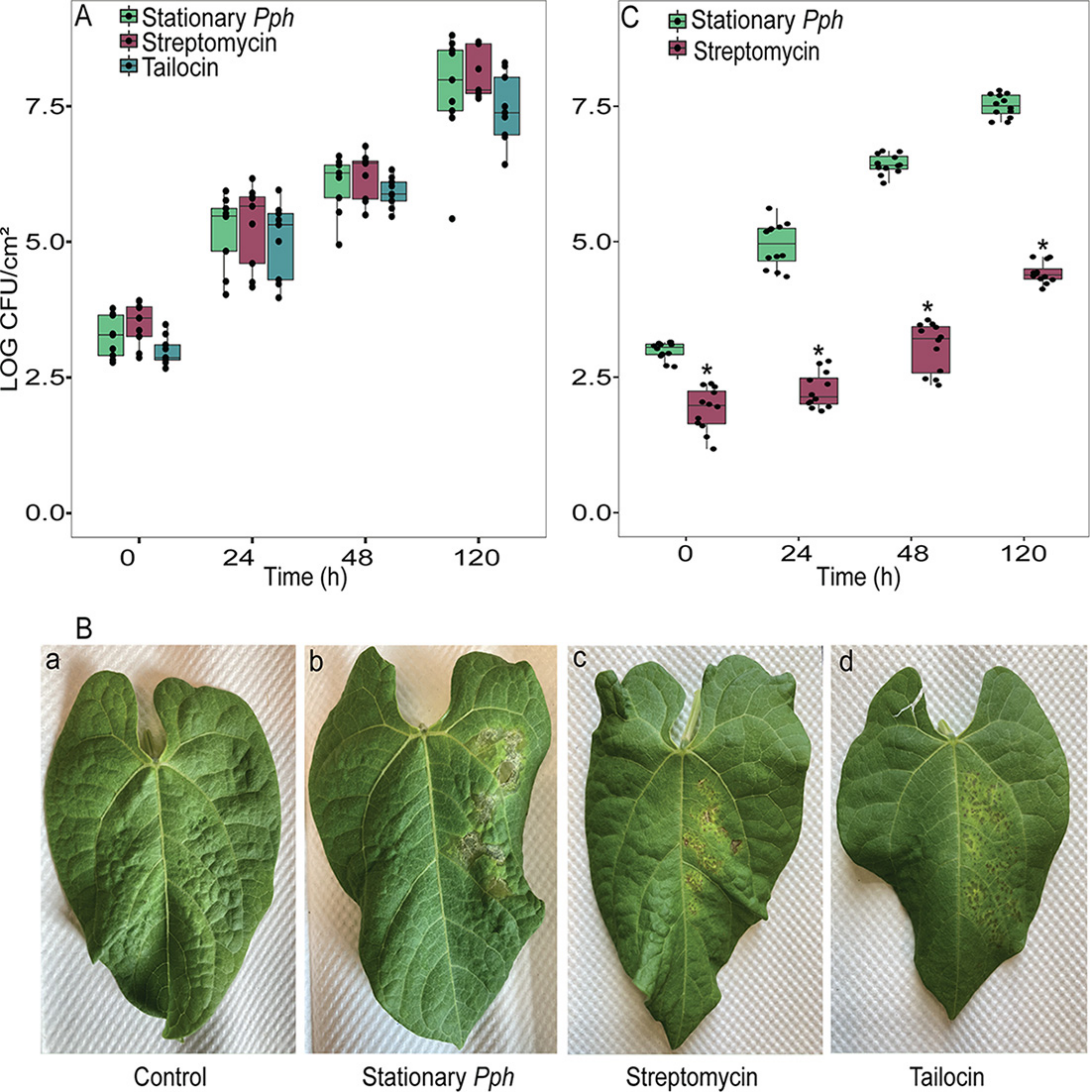
Colonization of host leaves by antimicrobial-treated *Pph*. Antimicrobial-treated populations of *Pph* colonize leaves at the same rate as untreated populations and form symptoms when the concentration is adjusted based on culturable cells (A, B) but exhibit delayed colonization when the concentration is adjusted based on RSG staining of cells (C). (A) Culturable *Pph* population growth in bean leaves after infiltration with untreated, streptomycin-treated, and tailocin-treated inocula that were adjusted based on CFU concentration. Inocula (200 μl) were syringe infiltrated into primary leaves of 15-day-old bean plants, and CFU were enumerated from leaf discs at the indicated time points. Dots represent the combined results of biological replicates from two independent experiments. No treatments showed significant differences within any time point. (B) Symptoms on bean leaves imaged 8 days after infiltration with untreated, streptomycin-treated, or tailocin-treated *Pph* and a water control. (C) *Pph* population growth in bean leaves after infiltration with untreated or streptomycin-treated inocula that were adjusted to contain equal proportions of redox-active cells, although proportions of culturable cells differed. Dots represent results of biological replicates from three independent experiments. Asterisks on the box plots represent values significantly different from those of stationary-phase *Pph* treatment within that time point (*P ≤ *0.05).

Because streptomycin persisters were associated with low activity, we hypothesized that streptomycin persisters might colonize the plant at a lower rate than the more active tailocin persisters. Sorting did not yield a sufficient number of cells to perform time point analysis, so we instead compared leaf colonization rates of treated and untreated cultures that had been adjusted to contain the same concentration of culturable cells (2.5 × 10^4^ CFU ml^−1^). Because streptomycin causes a vast decline in culturable cells, the streptomycin-treated inoculum contained roughly 700-fold more RSG-staining cells than the untreated inoculum. The tailocin inoculum contained a number of RSG-staining cells similar to that of the untreated inoculum but a far greater number of permeabilized cells. Despite differing viable population sizes and physiologies, there was no significant difference between the colonization rates of streptomycin- and tailocin-treated cultures in bean leaves and untreated cells (Fig. 5A). Streptomycin- and tailocin-treated cultures were able to cause symptoms of leaf spot and chlorosis at 8 days (Fig. 5B). Anecdotally, we observed that lesions seemed to appear a day earlier in the untreated inoculum and to coalesce to a greater degree by day 8 (Fig. 5B), but we did not inoculate enough leaves to perform quantitative assessment of symptoms. This finding indicates that the distinct physiological states of streptomycin and tailocin persisters do not delay their ability to colonize a susceptible host. It also suggests that in the streptomycin-stressed inoculum, the large populations of high-redox nonculturable cells may not make a significant contribution to early infection, or at least not enough to speed colonization of a susceptible host.

To test the last hypothesis, we performed a second experiment in which streptomycin-treated and untreated cultures were adjusted to contain the same proportion of redox-active cells, regardless of intensity or culturability. Inoculum adjustments were based on microscopic observations of mean RSG staining from Fig. 2. In this experiment, both inocula contained 7 × 10^4^ visibly RSG-staining cells per ml, but the untreated culture contained an estimated 450-fold-greater concentration of culturable cells than the streptomycin-treated inoculum. The streptomycin-treated population started growing in the leaf much more slowly than the untreated inoculum, with the population increasing only after a 2-day lag (Fig. 5C). This further supports the hypothesis that in a physiologically heterogeneous antibiotic-stressed *Pph* population, the high-redox unculturable cells do not significantly contribute to early host colonization.

### Streptomycin eradicates *Pph* persisters of tailocin.

**FIG 6:**
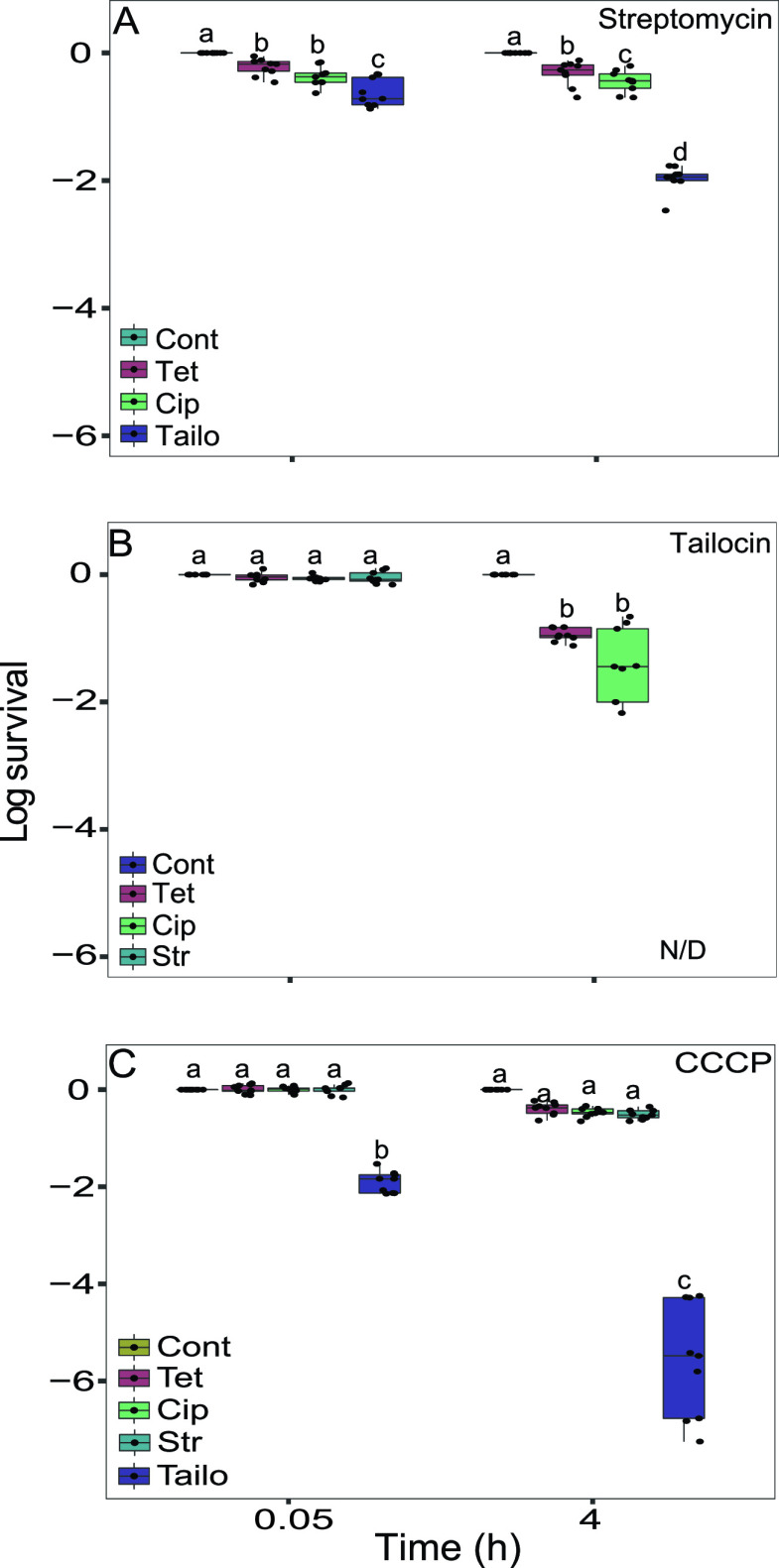
Tailocin effectiveness in eliminating streptomycin persisters and vice versa. (A to C) Survival of *Pph* persisters after sustained treatment with streptomycin (A), tailocin (B), and CCCP (C) and after being washed and exposed to a second antimicrobial treatment. Stationary-phase *Pph* organisms were treated, washed, and then treated with 5× MIC of the secondary antibiotic (5× MIC = 7.7 μg ml^−1^ for both ciprofloxacin and tetracycline). Cells were washed and enumerated immediately after the secondary treatment (0.05 h or 3 min) and at 4 h. Dots represent results from nine biological replicates collected in three independent experiments on different days. Letters denote statistical groups (*P* ≤ 0.05). N/D indicates a treatment from which no culturable cells were detected in any replicate.

Having determined that *Pph* tailocin persisters exist in a distinct physiological state from streptomycin persisters, we next hypothesized that streptomycin could eliminate tailocin persisters and vice versa. Antibiotic persisters often exhibit multidrug tolerance, so we also asked whether tailocin persisters could be eliminated by two other antibiotics, the bacteriostatic translational inhibitor tetracycline or the DNA replication inhibitor ciprofloxacin. Cross-survival rates of streptomycin persisters were first tested with a sequential treatment of tailocin, tetracycline, or ciprofloxacin. Thirty to 70% of the streptomycin-treated persistent population remained culturable after tetracycline or ciprofloxacin treatment, while only 1% remained culturable after tailocin treatment (Fig. 6A). Conversely, when tailocin persisters were washed and treated with tetracycline or ciprofloxacin, means of 7.2 and 11.5% remained culturable after treatment, respectively, while no colonies could be recovered after streptomycin treatment (Fig. 6B). To determine how this compares to rates of whole-population survival after exposure to these antibiotics, we treated stationary-phase *Pph* cultures with tetracycline or ciprofloxacin alone and measured CFU recovery at 6.5% ± 2.3% and 0.06% ± 0.02% of the initial population, respectively. Thus, compared to untreated *Pph* cells, tailocin persisters exhibited no survival in the presence of streptomycin, a survival rate similar to that of tetracycline, and a 178-fold-increased rate of survival in the presence of ciprofloxacin. CCCP-treated cells were also highly multidrug tolerant, but fewer than 0.1% survived tailocin treatment (Fig. 6C).

To further check for elimination of viable unculturable cells, we concentrated and microscopically examined the streptomycin-treated tailocin persisters and found that all cells stained with PI only or were unstained (Fig. S8). No category 1, 2, or 4 cells were observed. To rule out the possibility of rare live cells reviving to colonize the host, bean leaves were inoculated with concentrated cultures after the combination treatment. No symptoms developed on leaves (Fig. S8), and no *Pph* colonies were recovered in leaves collected immediately after inoculation or at days 1 to 5. In summary, cells surviving antibiotic and CCCP treatments have a high propensity to survive treatment with other antibiotics but are mostly eliminated by tailocin. Streptomycin treatment is highly effective at eliminating culturable tailocin persisters, while tetracycline and ciprofloxacin are less effective.

10.1128/mBio.00161-21.8 FIG S8 (A, B) Stationary-phase *Pph* cultures treated with tailocin and streptomycin, washed and stained with RSG, PI, and Hoechst 33342, and observed under a fluorescence phase-contrast microscope before treatment (A) and after treatment, followed by a 50× concentration (B). Scale bar = 20 μm. (C, D) Bean leaves infiltrated with a water control (C) and *Pph* cultures sequentially treated with tailocin and streptomycin (D). Note that the leaf image in panel C is also shown as the negative-control image in Fig. 5B, as these inoculations were performed on the same day under the same conditions. Download FIG S8, TIF file, 2.9 MB. Copyright © 2021 Patel et al. 2021 Patel et al. https://creativecommons.org/licenses/by/4.0/ This content is distributed under the terms of the Creative Commons Attribution 4.0 International license.

## DISCUSSION

Membrane-disrupting treatments have long shown promise for disease control and for eradication of antibiotic persisters, and there remains an enormous trove of membrane-disrupting biological compounds still to be discovered (37). Understanding the basis and management of population-level tolerance to these compounds will be important to maximize their efficacy. Here, a study of physiological heterogeneity in the model plant pathogen *Pph* demonstrated that tailocin and streptomycin treatments have vastly different physiological consequences. The small fraction of culturable cells surviving each treatment exhibited distinct redox phenotypes and corresponded closely with the fractions capable of causing infection. The study shows that that streptomycin and tailocin may be a potent combination treatment for sterilization of *Pph* cultures, including the elimination of viable nonculturable cells. The study also links redox states to heterogeneous persistence and virulence phenotypes, which may inform the search for associated mechanisms and markers.

Tailocins are triggered by the recognition of specific lipopolysaccharides (LPSs) on the target cell surface, after which the tail is driven into the membrane using energy stored in its contractile structure (38). The consequences are rapid membrane depolarization and ATP depletion, as well as transcriptional and translational arrest, from as little as one particle per target cell (39, 40). How, then, can persisters survive and maintain redox activity? The finding of nondormancy is consistent with an active mechanism of tailocin survival, and the rapid time frame of killing suggests that this trait is expressed in a portion of the planktonic population rather than being tailocin induced. The metabolic activity of tailocin persisters is reminiscent of conditional tolerance to the membrane-destabilizing peptide colistin in Pseudomonas aeruginosa and other animal pathogens. Colistin tolerance was associated with increased expression of Pmr proteins that modify LPS to reduce colistin affinity, meaning that only transcriptionally active cells avoid membrane destabilization (29, 30, 41). Tailocin sensitivity is also linked to the compositions of LPSs (42), which can vary according to changes in environment and gene expression (43). We recently found that an LPS cluster gene of unknown function affects the frequency of tailocin persisters without impacting tailocin susceptibility or host fitness (32). One hypothesis consistent with our findings is that persistence derives from active tailocin avoidance, potentially through population heterogeneity in tailocin recognition targets or other susceptibility factors. Further molecular analysis of the persistent population will be needed to pinpoint the underlying mechanisms. The streptomycin sensitivity and ciprofloxacin tolerance that we observed in tailocin persisters also echoes recent work on colistin, which was found to be much more efficient in eliminating aminoglycoside persisters than eliminating ciprofloxacin persisters in E. coli (11). The authors hypothesized that colistin works synergistically with aminoglycosides due to the membrane damage exerted by both treatments. Our study suggests that aminoglycoside synergy, and perhaps fluoroquinolone cross-tolerance, may be common themes of LPS-destabilizing antimicrobials.

This study employed complementary methods of flow cytometry and fluorescence microscopy to profile antimicrobial-induced changes in *Pph* populations. Microscopy was useful in distinguishing intermediate viability categories, demonstrating that a third of redox-active cells were permeable to PI after streptomycin treatment. Membrane-damaged live cells were previously found to self-repair and resuscitate from VBNC populations of *Pseudomonas* and *Shewanella* spp. (44) and have been observed in nonstressed growing populations of other bacteria (45). This study indicates that DRAQ7 provides a more confident indication of fatal membrane damage for *Pseudomonas*. Streptomycin also induced a high proportion of “category 4” cells, retaining intact membranes and increased roundness but no redox signal, a state similar to one associated with persisters, VBNC cells, and newly dead cells of E. coli (7, 9). We hypothesize that category 4 includes the fraction associated with streptomycin persistence in sorting experiments, which similarly lacked redox signal above that of dead cells. Tailocin reduced the proportion of category 4 cells in *Pph* cultures by 99.9% (Fig. 2E), consistent with its ability to target dormant cells. However, the abundance of cells in each microscopy category exceeded the abundance of persisters, illustrating that each phenotype contains heterogeneity and that persistence levels cannot be anticipated by staining phenotype alone.

Streptomycin treatment shifted the majority of the intact *Pph* population to a state of increased RSG staining intensity. Antibiotics stimulate the production of reactive oxygen species (ROS) in bacteria (46), and antibiotic-induced increases in RSG intensity were recently associated with an accumulation of ROS-protective reductases in Campylobacter jejuni (47). Thus, we suspect that the high-redox fraction in *Pph* cells similarly reflects a reductase response to intracellular ROS production, although ROS-specific methods would be needed to determine this conclusively. If so, the nonculturability of this fraction may be consistent with findings that ROS avoidance is a marker of persistence and postantibiotic culturability (48, 49), although in our study, even the fraction of moderate RSG intensity was unculturable after streptomycin removal. All intact cells are counted as live or VBNC cells using common permeability-based quantification methods (50), but the high-redox cells that we observed are distinct from the VBNC cells induced by long-term starvation, reported as being dormant and persister-like (6, 7). This study demonstrates that the large live-but-unculturable fraction does not revive in a susceptible host or greatly contribute to short-term infection in a mixed population.

Even in the absence of antimicrobial treatment, the fraction of *Pph* cells with the strongest redox signal did not cause symptoms in the host. In recent years, it has become clear that pathogenicity to plants is often a heterogeneously expressed trait, with essential virulence factors produced in a population-bistable manner in P. syringae and other plant pathogens (51, 52). The virulent state is associated with a suppression of genes involved in active growth processes (53, 54). This study links one avirulent *Pph* subpopulation to an increased metabolic rate. It is striking that 5 h of exposure to a long-relied-upon disease control treatment did not greatly reduce the number of intact and active cells but rather shifted much of the population toward a noninfectious state. A more complete understanding of how antibacterial treatments affect pathogen physiology, both in the lab and in the field, will be essential in tailoring disease control strategies that are more effective in reducing the pathogen inoculum.

## MATERIALS AND METHODS

### Bacterial strains, plant lines, and culture conditions.

Tailocin was prepared from cultures of P. syringae pv. *syringae* strain B728a. Experiments were performed using P. syringae pv. *phaseolicola* strain 1448A (*Pph*). Cultures were grown from a single colony in King’s medium B (55) at 28°C, with 200-rpm shaking, unless otherwise indicated. Common bean (Phaseolus vulgaris) variety Kentucky Wonder (Seed Savers’ Exchange, Decorah, IA) plants were grown in disposable plastic pots (8 by 6 cm and 8-cm deep) in Pro-Mix growing BX Mycorrhizae medium and maintained at 23°C with 70% relative humidity for a 16-h day length in a Conviron growth chamber. For bean pod inoculations, plants were grown in a greenhouse (24 to 26°C) in large pots (12-cm diameter and 12-cm deep). Pods were collected from 50- to 55-day-old bean plants.

### Tailocin preparation.

Tailocin was prepared and quantified from supernatants of P. syringae pv. *syringae* B728a as previously described (34, 56). Overnight cultures of B728a were diluted 1:100 in King’s B medium and grown for 3 h at 28°C, and tailocin production was induced by the addition of mitomycin C (MP Biomedicals LLC, Solon, OH) to a concentration of 0.5 μg ml^−1^. After a 24-h induction, supernatants were collected by centrifugation. Residual live cells were killed by treating the supernatant with chloroform. The aqueous phase was collected by centrifugation and then amended with NaCl and polyethylene glycol 8000 (PEG 8000) to final concentrations of 1 M and 10%, wt/vol, respectively. After 1 h of incubation on ice, the supernatant mixture was centrifuged at 16,000 × *g* for 30 min at 4°C. The resulting tailocin pellet was dissolved in 10 mM Tris (pH 7.0) and 10 mM MgSO_4_. Residual PEG 8000 was removed by two extractions with equal volumes of chloroform. The activity of prepared tailocin was evaluated by spotting 5-μl serial dilutions onto soft agar overlay plates seeded with *Pph*. Tailocin activity is expressed in activity units (AU) derived from the highest dilution factor resulting in a visible inhibition zone (57).

### MICs.

The MICs of streptomycin (MP Biomedicals LLC, Solon, OH), tetracycline (MP Biomedicals LLC, Solon, OH), and ciprofloxacin (Acros Organics, Fair Lawn, NJ) for *Pph* were determined by evaluation of turbidity using a previously described method (58), with some modifications. An overnight culture of *Pph* was diluted to an optical density at 600 nm (OD_600_) of 0.1 in King’s B medium, and 20 μl of the cell suspension was added to 180 μl of King’s B medium amended with antibiotics to achieve final antibiotic concentrations of 25, 12.5, 6.25, 3.12, 1.56, 0.78, 0.39, 0.19, and 0 μg ml^−1^ in a 200-μl volume. Growth was assessed by measuring the OD_600_ over 20 h using an absorbance plate reader (Bio-Tek). The MIC of each antibiotic was the lowest concentration at which no increase in turbidity was measured across at least three independent cultures. The MIC of tailocin was similarly determined in activity units (AU), starting with nine 1:2 serial dilutions of the initial tailocin preparation.

### Killing curve of *Pph* after treatment with streptomycin and tailocin.

To prepare stationary-phase cultures of *Pph*, a single colony was inoculated into 5 ml King’s B broth, grown for 20 h at 28°C, diluted 1:100, and grown for 18 h (typically to an OD_600_ of 1.3) or 4 days. To prepare log-phase cultures, a 20-h culture was diluted 1:50 in King’s B medium and incubated for 2.5 h (OD_600_ = 0.15). To perform killing curve experiments, streptomycin was added to the cultures to reach a concentration of 16 μg ml^−1^ (5× MIC), followed by a shaking incubation at 28°C for 5 h. One-milliliter samples were collected prior to streptomycin addition (T0) and hourly for 5 h. Samples were centrifuged 2 min at 13,000 rpm, resuspended two times in sterile saline (0.8% NaCl), and enumerated by dilution plating on King’s B agar. Colonies were counted at 48 h.

Tailocin killing curves were generated as previously described (32), with modifications. Log-phase, stationary-phase, or 4-day-old *Pph* cultures were diluted to an OD_600_ of 0.1 in 0.8% NaCl, and tailocin was added to a concentration of 250 AU ml^−1^, which represents 5× MIC for *Pph*. Samples were removed before and immediately after the addition of tailocin and then each hour for 4 h. Samples were washed twice with saline and enumerated by serial dilution. Addition of the first wash was typically completed in under 3 min from sample collection; therefore, the sample removed immediately after tailocin addition was termed the T0.05 sample.

The antibiotic susceptibilities of tailocin and streptomycin persisters were confirmed by measuring the persistence level of cultures grown from surviving colonies. Three colonies were selected at random from the final time point of each experiment in Fig. 1 and used to initiate three new cultures that were grown to stationary phase (20 h) in King’s medium B. *Pph* cells were reexposed to the initial tailocin or streptomycin treatment for 4 h or 5 h, as described above, to confirm that the proportion of survivors had not changed.

### Microscopic cell physiology analysis of *Pph*.

Staining of *Pph* with RedoxSensor green (RSG) and propidium iodide (PI) was performed using the BacLight RSG vitality staining kit (with additional staining with Hoechst 33342 dye [both from Thermo Fischer Scientific, USA]). To determine the impact of the combined fluorescence dyes on the culturability of *Pph*, 10 μl of 1 mM RSG, 10 μl of 20 mM PI, and 15 μl of 1 μM Hoechst 33342 were added to 1 ml of a stationary-phase *Pph* culture and incubated in the dark at room temperature for 30 min. Stained cells and unstained control cultures were washed twice and diluted in 0.8% NaCl and plated onto King’s B agar. Numbers of CFU from stained and unstained cells were recorded. The same procedure was used to assess the impact of DRAQ7 and RSG costaining on the culturability of *Pph*. Ethanol-killed cell populations were generated by resuspending stationary-phase *Pph* in phosphate-buffered saline (PBS) at an OD_600_ of 0.2, adding an equal volume of 70% ethanol for 15 min, and washing the cells twice with PBS prior to staining them.

*Pph* cultures at log and stationary phases were treated with streptomycin (5 h) or tailocin (4 h) as described above. Total cells were enumerated by hemocytometer counting under a phase-contrast microscope. Culturable cells were enumerated by dilution plating after the cells were washed twice with saline (0.8% NaCl). Agarose pads (1.5%) were prepared on glass slides as previously described (59). Treated and untreated cells (10 μl) were amended with 0.1 μl RSG (1 mM), 0.1 μl PI (20 mM), and 0.15 μl Hoechst 33342 (1 mM) and incubated for 10 min in the dark. One microliter of culture was placed on the middle of the agarose pad. Images were collected using a Zeiss Axio Imager M1 fluorescence microscope within 20 min of placement of the cells on the pad. Multichannel images were captured using FITC (fluorescein isothiocyanate), rhodamine, and DAPI (4′,6-diamidino-2-phenylindole) filter sets in Zen 2.6 (blue edition) software. For each of four independent experiments, 10 fields were imaged across at least three different slides per treatment. All cells were counted in each image, totaling a range of 1,000 to 1,700 cells for each treatment and time point in each experiment, except for the tailocin 4-h time point, for which there were 650 to 700 cells in the 10 fields. Single cells were classified into five staining categories (green/blue, red/green/blue, red/blue, blue, or unstained) by visual comparison of the same cell under three different channels and in phase contrast. Cell counts were recorded by clicking on each cell using the Cell Counter plugin in Fiji (60). The intensity and roundness of selected cells were measured using the MicrobeJ plugin in Fiji (61).

### Flow cytometry.

The physiological states of streptomycin- and tailocin-treated cultures were evaluated through redox and cell integrity staining, followed by flow cytometric analysis. Four treatments were selected for flow cytometry: stationary-phase *Pph* culture, stationary-phase culture treated with streptomycin or tailocin as described above, or stationary-phase culture treated with 100 μg ml^−1^ carbonyl cyanide *m*-chlorophenylhydrazone (CCCP; Sigma-Aldrich) for 3 h. Stationary-phase cells were washed in saline and resuspended in saline to an OD_600_ of 0.2 before treatment. After treatment, *Pph* cells were washed twice with PBS, and then the bacterial suspension was stained with RSG (1 ml bacterial suspension was incubated with 1 μl of RSG working solution [1 mM] and 3 μl DRAQ7 working solution [3 mM] for 30 min in the dark at room temperature). Stained samples were analyzed on the BD FACSAria-II at the Yale Flow Cytometry Facility (Yale School of Medicine, New Haven, CT, USA). Excitation wavelengths were 488 nm and 633 nm. Fluorescence was collected with a 530/30-bandpass filter for RSG and with a 710/25-bandpass filter for DRAQ7. Data analysis was performed using BD FACSDiva 8.0.1 and FlowJo 10.6.2.

### Fluorescence-assisted cell sorting (FACS), culturability, and virulence tests.

During flow cytometry, the forward scatter/side scatter (FSC-A/SSC-A) dot plots of each isolate were used to define the total bacterial population. Doublets and debris were excluded via the contour and dot plots. Based on 50,000 sorted events, regions of green and red fluorescence intensity defined by stationary-phase, ethanol-killed, and unstained *Pph* cells were used to define three gates associated with bright redox signal intensity (G1), medium intensity (G2), and low intensity (G3) among intact cells. Cultures were aseptically sorted into tubes until 10^7^ events were collected or, for low-density gates, until the entire suspension was sorted. Collected fractions were adjusted to a final concentration of 0.1% sterile peptone buffer, a common diluent used in *Pph* enumeration (62), in a final volume of 1 ml. Isolated dots outside the polygon were not included in the analysis.

For culturing studies, sorted fractions were centrifuged at 13,000 rpm for 2 min, and pellets were resuspended in 50 μl PBS. Twenty-microliter aliquots of the suspension were serially diluted, and 5 μl was spotted in triplicate on King’s B agar plates. Colonies were enumerated after 48 h of incubation at 28°C.

Sorted fractions were inoculated on detached bean pods according to the method of Bozkurt and Soylu (63). In brief, mature bean pods (from 50-day-old plants) were collected, washed in distilled water, surface sterilized in 70% ethanol, and pierced using sterile 10-μl pipette tips. Thirty microliters of the 50-μl concentrated sorted fraction was placed on the wound. Inoculated pods were stored in sterile plastic containers lined with moist Whatman filter paper and incubated in a 28°C chamber. Disease symptoms were recorded at 5 days after incubation.

### *Pph* inoculation to bean plants.

Fifteen-day-old bean plants were inoculated with untreated, streptomycin-treated, tailocin-treated, or streptomycin- and tailocin-treated stationary-phase cultures of *Pph*. Treated cultures were prepared using the methods and ending time points described for stationary-phase killing curves, with streptomycin-tailocin combination treatments incubated for 4 h. Untreated cultures were diluted in PBS to an OD of 0.0001, and single-antibiotic-treated cultures were diluted to achieve the same concentration of culturable cells as the untreated inoculum (Fig. 6A) or the same concentration of RSG-staining cells as the untreated inoculum (Fig. 6B). Concentration adjustments were made based on observations from repeated prior experiments. Two hundred microliters of inoculum was used to infiltrate the underside of the primary leaves of bean plants using 1-ml BD syringes. Samples from infiltrated areas were collected at 0, 1, 2, and 5 days postinfection (dpi) using a 1-cm cork borer. Leaf discs were collected into a 1.5-ml tube containing 200 μl 10 mM MgCl_2_ and homogenized using disposable pellet pestles (Fischer Scientific). Homogenates were serially diluted on King’s B agar supplemented with 50 μg ml^−1^ nalidixic acid, to which *Pph* is genetically resistant, and CFU were enumerated after 48 h of incubation at 28°C.

### Antibiotic cross-tolerance experiments.

To measure the cross-tolerance of streptomycin-tolerant *Pph* cultures against other antibiotics and tailocin, stationary-phase *Pph* cultures were washed and resuspended to an OD_600_ of 0.1 in saline and treated with 16-μg ml^−1^ streptomycin for 5 h. Cells were washed twice, resuspended in saline to remove streptomycin, and then treated for 4 h with tailocin (250 AU ml^−1^), tetracycline (8 μg ml^−1^), or ciprofloxacin (8 μg ml^−1^) before being washed again and serially diluted. Low-CFU samples were resuspended in a reduced volume of saline (50 μl) after the final wash. The same procedure was followed to determine the tolerance level of CCCP-treated or tailocin-treated stationary-phase cultures to other antibiotics, with the following modifications: cultures were treated with CCCP at a concentration of 100 μg ml^−1^ for 3 h or with 250 AU of tailocin for 1 h.

### Statistical analysis.

Differences in total numbers and numbers of culturable cells were assessed using a Student *t* test (two-tailed distribution with two-sample, equal-variance calculations). Multiple comparisons were performed with one-way analysis of variance (ANOVA). Means were separated using Tukey’s honestly significant difference test at a *P* of 0.05. Statistical analyses were performed in R version 4.0.3.

### Data availability.

Images of fields used to generate Fig. 2, Fig. S2, and Tables S1 and 2 are available on figshare (https://doi.org/10.6084/m9.figshare.13526240.v1).
