# Supplementary figures and images for: Pseudomonas syringae pv. phaseolicola Uses Distinct Modes of Stationary-Phase Persistence To Survive Bacteriocin and Streptomycin Treatments
Source: mBio. 2021 Apr 13;12(2):e00161-21. doi: 10.1128/mBio.00161-21 (PMC8092213; doi:10.1128/mBio.00161-21)

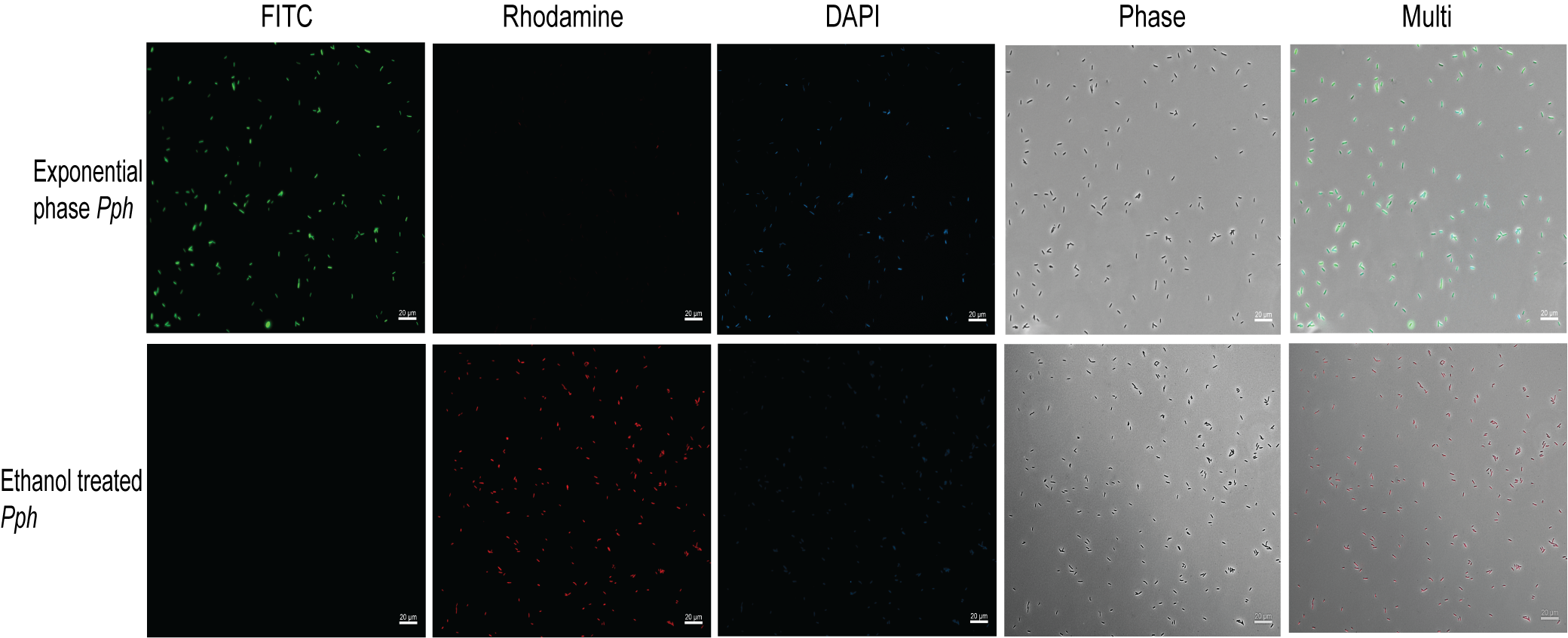

Supplement: FIG S3 [file mBio.00161-21_sf003.tif]

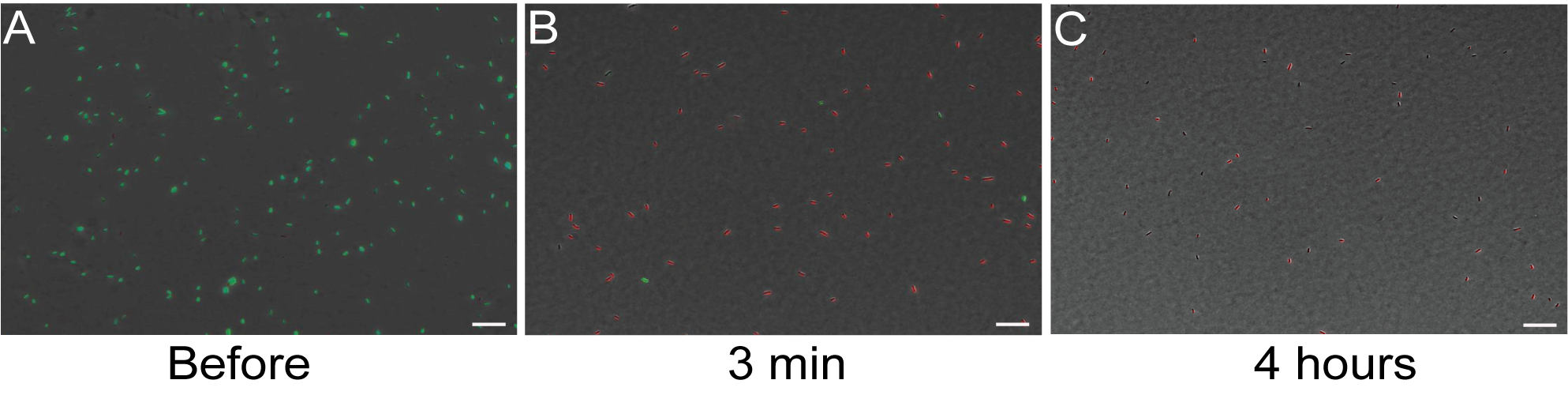

Supplement: FIG S4 [file mBio.00161-21_sf004.tif]

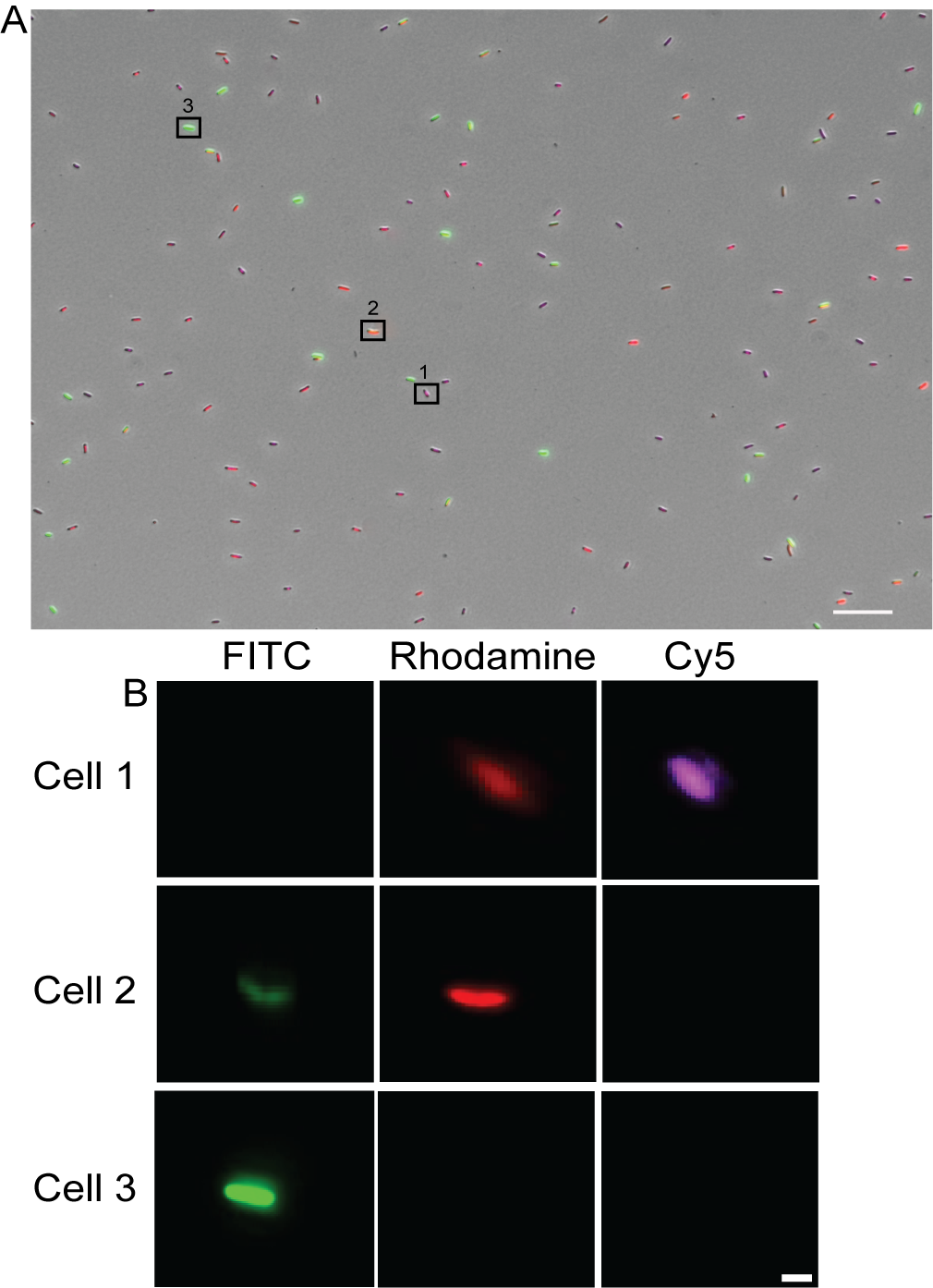

Supplement: FIG S6 [file mBio.00161-21_sf006.tif]

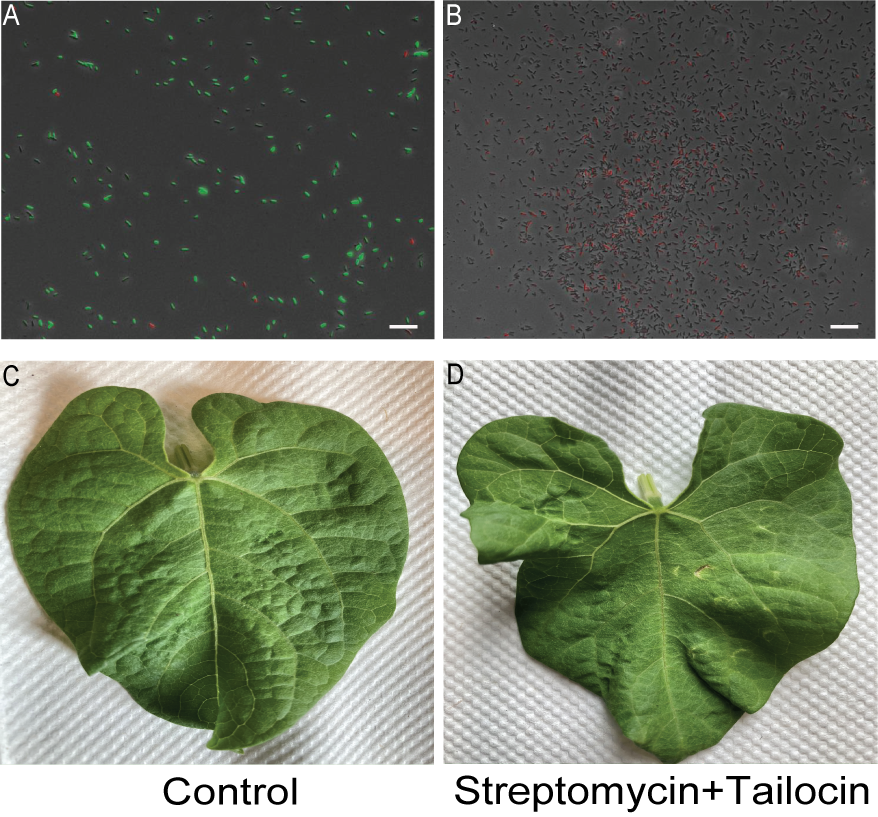

Supplement: FIG S8 [file mBio.00161-21_sf008.tif]
