# Supplementary material for: Pseudomonas syringae pv. phaseolicola Uses Distinct Modes of Stationary-Phase Persistence To Survive Bacteriocin and Streptomycin Treatments
Source: mBio. 2021 Apr 13;12(2):e00161-21. doi: 10.1128/mBio.00161-21 (PMC8092213; doi:10.1128/mBio.00161-21)
Supplement: TABLE S1 [file mBio.00161-21_st001.docx]

Table S1. Percentage of imaged log phase and stationary phase *Pph* cells in five fluorescent staining categories before and after antimicrobial treatment.

| Treatment | Category 1  (RSG) | Category 2  (RSG + PI) | Category 3  (PI) | Category 4 (Hoescht) | Category 5 (Unstained) | Total RSG |
| --- | --- | --- | --- | --- | --- | --- |
|  | Stationary Phase | | | | | |
| *Pph* T0 | 83.2±8 *^a^* | 1.7±3 | 8.2±3 | 3.3±3 | 3.3±3 | 84.9±9^b^ |
| *Pph* + Str | 24.9±11 | 19.2±9 | 24.1±10 | 25.4±7 | 6.2±3 | 44.1±14 |
| *Pph* + Tail | 3.1±3 | 17.4±12 | 71.1±14 | 0.4±1 | 7.8±5 | 20.5±12 |
|  | Log Phase | | | | | |
| *Pph* T0 | 85.9±5^c^ | 7.7±2 | 1.70±3 | 3.1±3 | 1.5±2 | 93.6±5 |
| *Pph* + Str | 36.9±13 | 14.4±9 | 19.9±13 | 24.6±15 | 4.1±4 | 51.3±16 |
| *Pph* + Tail | 1.1±3 | 1.3±2 | 34.3±13 | 0.1±0.3 | 63.1±14 | 2.4±4 |

a. Values represent the mean percentage ±SD of stationary phase *Pph* cells in each category. T0 values represent combined T0 values from eight streptomycin and tailocin experiments in depicted Fig. 2D and 2E.

b. Total vitality estimate, or percentage of cells staining with RSG, calculated as the sum of Categories 1 and 2.

c. Represents the mean percentage ±SD of log phase *Pph* cells in each category. T0 values represent combined T0 values from eight streptomycin and tailocin experiments in depicted Fig. S5D and E.
