# Supplementary material for: Pseudomonas syringae pv. phaseolicola Uses Distinct Modes of Stationary-Phase Persistence To Survive Bacteriocin and Streptomycin Treatments
Source: mBio. 2021 Apr 13;12(2):e00161-21. doi: 10.1128/mBio.00161-21 (PMC8092213; doi:10.1128/mBio.00161-21)
Supplement: TABLE S2 [file mBio.00161-21_st002.docx]

Table S2. Percentage of imaged cells staining with RSG, DRAQ7, or Hoescht only in microscopic analysis of stationary phase *Pph* before or after antimicrobial treatment.

| Treatment |  | | Stationary Phase | | | |
| --- | --- | --- | --- | --- | --- | --- |
|  | RSG | RSG+DRAQ7 | | DRAQ7 | Hoechst | Unstained |
| *Pph* T0 | 87.5^a^ | 0 | | 8.9 | 1.7 | 1.8 |
| Str + *Pph* | 46.4 | 0 | | 28.8 | 18.3 | 6.3 |
| Tail + *Pph* | 4.1 | 0 | | 64.1 | 0.3 | 31.3 |

a. Values represent the percentage of all *Pph* cells in each staining category in a single experiment. Cells were counted from 10 images taken from across four slides.
